# Impact of experimental colitis on mitochondrial bioenergetics in intestinal epithelial cells

**DOI:** 10.1038/s41598-022-11123-w

**Published:** 2022-05-06

**Authors:** Luke Goudie, Nicole L. Mancini, Timothy E. Shutt, Graham P. Holloway, Chunlong Mu, Arthur Wang, Derek M. McKay, Jane Shearer

**Affiliations:** 1 Department of Biomedical Engineering, Schulich School of Engineering, University of Calgary, Alberta, Canada; 2 Gastrointestinal Research Group and Inflammation Research Network, Department of Physiology and Pharmacology, Calvin, Joan and Phoebe Snyder Institute for Chronic Diseases, Cumming School of Medicine, University of Calgary, Alberta, Canada; 3 Alberta Children’s Hospital Research Institute, Hotchkiss Brain Institute, Departments of Medical Genetics and Biochemistry and Molecular Biology, Cumming School of Medicine, University of Calgary, 3330 Hospital Drive NW, HMRB 228, Alberta, Canada; 4 Department of Human Health and Nutritional Sciences, University of Guelph, Guelph, ON Canada

**Keywords:** Gastrointestinal models, Mechanisms of disease, Mitochondria, Biophysical methods, Mitochondria, Biochemistry, Physiology

## Abstract

Intestinal homeostasis is highly dependent on optimal epithelial barrier function and permeability. Intestinal epithelial cells (IEC) regulate these properties acting as cellular gatekeepers by selectively absorbing nutrients and controlling the passage of luminal bacteria. These functions are energy demanding processes that are presumably met through mitochondrial-based processes. Routine methods for examining IEC mitochondrial function remain sparse, hence, our objective is to present standardized methods for quantifying mitochondrial energetics in an immortalized IEC line. Employing the murine IEC^4.1^ cell line, we present adapted methods and protocols to examine mitochondrial function using two well-known platforms: the Seahorse Extracellular Flux Analyzer and Oxygraph-2 k. To demonstrate the applicability of these protocols and instruments, IEC were treated with and without the murine colitogenic agent, dextran sulfate sodium (DSS, 2% w/v). Profound impairments with DSS treatment were found with both platforms, however, the Oxygraph-2 k allowed greater resolution of affected pathways including short-chain fatty acid metabolism. Mitochondrial functional analysis is a novel tool to explore the relationship between IEC energetics and functional consequences within the contexts of health and disease. The outlined methods offer an introductory starting point for such assessment and provide the investigator with insights into platform-specific capabilities.

## Introduction

The maintenance of the intestinal barrier is a highly energetic process. Composed of a single layer of epithelial cells, tight junctions, and mucosal secretions, optimal barrier function is essential to preventing pathogen migration into the mucosa and subsequent inflammation. The vast majority of the energy required to maintain the barrier is derived from mitochondria^1^. This has been readily demonstrated, since inhibition of mitochondrial oxidative phosphorylation and the resulting ATP depletion, leads to reduces intestinal barrier function and increased permeability^2^. The opposite also holds true as mice exhibiting mitochondrial DNA polymorphisms that improve oxidative capacity and subsequent ATP generation are protected from experimental colitis^3^.

Energy generation in intestinal epithelial cells (IEC) is multifaceted and varies depending on location, stage of maturation, nutrient availability, and the presence of disease. Mitochondrial dysfunction is also a prominent feature of many chronic disease states including inflammatory bowel disease (IBD). Colonocytes isolated from patients with active or quiescent ulcerative colitis (UC), exhibit reduced rates of butyrate metabolism, and a compensatory increase in glucose and glutamine metabolism^4^. These observations have been replicated in biopsies from patients with UC or Crohn’s disease during quiescent or active disease states^5,6^. Dysfunctional mitochondria also play a pivotal role in reactive oxygen species generation^7^, triggering inflammation^8^ and apoptosis^9^. Therefore, the case can be made that the study of mitochondria is of paramount importance when considering the role of the enteric epithelium in health and disease^10–12^.

Despite convincing and substantive evidence relating mitochondrial bioenergetic processes to colitis and disease, there remains a paucity of information and methods to conveniently and specifically assess mitochondrial respiratory function in IECs. Addressing this gap, we herein demonstrate the use of two platforms to interrogate oxygen consumption and subsequently mitochondrial function in IECs. The Seahorse Extracellular Flux Analyzer (Seahorse XF, Agilent Technologies, U.S.) platform is often utilized to screen for general differences in respirometric function between treatments. Administered as a kit, it is relatively easy to use for the novice investigator and allows for high throughput experimental trials. In contrast, the Oxygraph-2 k platform (O2k, Oroboros Instruments, Austria) is a customizable, lower throughput platform with the ability to interrogate specific substrates and sites of mitochondrial respiration.

This study describes each methodology, provides expected experimental results, and considerations for the design and execution of mitochondrial functional assessments in IEC. As a proof of principle study, healthy control IECs were compared to those exposed to dextran sulfate sodium (DSS), a common colitis instigator used in experimental murine colitis models^13^ in both platforms. Consistent with reports of mitochondrial dysfunction in DSS murine colitis models^14,15^, our results similarly show significant disparities in mitochondrial function after DSS exposure.

## Results

### IEC quantification and optimization for metabolic assessment

Detailed respirometric measures are routinely performed in tissues, cells, and isolated mitochondria^16^. Such assays have led to numerous insights into the role of mitochondria in health and disease. Specific and detailed protocols for conducting respirometric analysis in IEC have recently been described^17,18^, but are not widely employed. This is particularly surprising, with the potential relevance of IEC energetics to the development or exaggeration of IBD and other enteropathies^4–12^. This study therefore sought to develop and discuss, step-by-step instructions to provide an introductory lens into using both the Oroboros O2k and Seahorse XF to study IEC^4.1^ mitochondrial function.

The cells employed are the murine intestinal epithelial cell line IEC^4.1^, a transformed but non-tumorigenic intestinal epithelial cell-derived from neonatal mice^19^. Changes in cell viability and cell growth were monitored by counts using trypan blue during passages and experiments were conducted on IEC^4.1^ cells between the passages of 50 and 65^19^. As a proof of principle experiment to demonstrate both respirometric platforms, we compared control cells with those treated with the murine colonic agent DSS (2%) for a period of 24 h. Of note, procedures also exist for freshly isolated IEC and are described elsewhere^17^.

### Optimization of metabolic assessment in the seahorse XF measurement system

Before proceeding with experiments, optimal cell numbers for each platform were determined. For the Seahorse XF system, we examined mitochondrial oxygen consumption rates (OCR) under different cell seeding densities (2 × 10^4^/well to 5 × 10^4^/well) and varying FCCP concentrations (0.25–1 µM) (Supplementary Fig. 1–2). Based on experimentally determined cell growth findings, as well as, both basal and ATP associated OCR measurements, the seeding density of 2 × 10^4^ cells/well was selected for use in Seahorse 24 well cartridges (Supplementary Fig. 1). In optimizing FCCP concentrations for the Seahorse platform, no differences were found in concentrations ranging from 0.25 to 1 µM (Supplementary Fig. 2). However, in optimizing FCCP concentration for the Oxygraph, FCCP induced maximal respiration was found to occur at concentrations ranging from 0.5 to 0.6 µM in both control and DSS cell populations (Supplementary Fig. 3A and B). Since FCCP induced OCR remained relatively similar across FCCP concentrations of 0.25–1 µM, we chose to use the concentration of 0.5 µM FCCP for future Seahorse experiments to maintain consistency with Oroboros O2k experiments. If using the 96 well plate format, this optimization will need to be repeated. These levels represented the mid-range of measures, allowing for both increases and decreases in respiration to be observed and are detailed in the methods. Assays were performed using serial titrations of 1.3 μM Oligomycin, 0.5 μM FCCP, a combination of 1.0 μM Rotenone and 1.0 μM Antimycin A. In addition, a complete list of chemicals and their respective actions on mitochondrial respiration are shown in Table 1. Before analysis, growth media was pipetted off to remove any dead cells during growth and fresh assay media was added. At the end of the experiment, both viable and dead cells were counted to normalize respirometric data.

**Table 1:** Reagents and their effect on mitochondrial electron transport chain complexes or metabolic pathways.

Chemical	Inhibitor/uncoupler	Relevance to mitochondrial oxidative phosphorylation
Adenosine diphosphate	Substrate	A reactant in the production of ATP by ATP synthase. When combined with other substrates, it allows oxidative phosphorylation substrate and mitochondrial complex capacities to be examined
Pyruvate	Substrate	NADH linked substrate, produced from the oxidation of glucose after glycolysis
Malate	Substrate	NADH linked substrate, produced during the TCA cycle
Butyrate	Substrate	Vital short-chain fatty acid for beta-oxidation by colonocytes. Must be added with malate to ensure no feedforward inhibition
Cytochrome C	Substrate	Electron carrier for CIII to CIV. Increased oxygen consumption rate in response to exogenous cytochrome c additions indicate outer mitochondrial membrane damage
Glutamate	Substrate	NADH linked substrate produced during glutaminolysis that feeds into the TCA cycle
Succinate	Substrate	FADH2 linked substrate, produced during the TCA cycle
N,N,N′,N′-tetramethyl-p-phenylenediamine (TMPD)	Substrate	Substrate for determining CIV activity. Must be paired with ascorbate to prevent auto-oxidation of TMPD by oxygen
Ascorbate	Substrate	Donates electrons to N,N,N',N'-Tetramethyl-p-phenylenediamine dihydrochloride (TMPD) and limits auto-oxidation by oxygen
FCCP/CCCP	Uncoupler(s)	Protonophore that uncouples mitochondrial oxidative phosphorylation. The uncoupling of oxidative phosphorylation with FCCP allows for the determination of a theoretical maximal mitochondrial capacity
Rotenone	Inhibitor	Inhibitor of mitochondrial complex I
Antimycin A	Inhibitor	Inhibitor of mitochondrial complex III. Completely inhibits mitochondrial respiration when paired with rotenone
Sodium azide	Inhibitor	Inhibitor of CIV
Oligomycin	Inhibitor	Inhibitor of ATP synthase
Digitonin	Permeabilizing agent	Mild detergent that allows for selective permeabilization of the cell membrane, but not typically the mitochondrial membrane, unless it is over titrated

### Impact of DSS on the seahorse XF cell mito stress test

**Figure 1:**
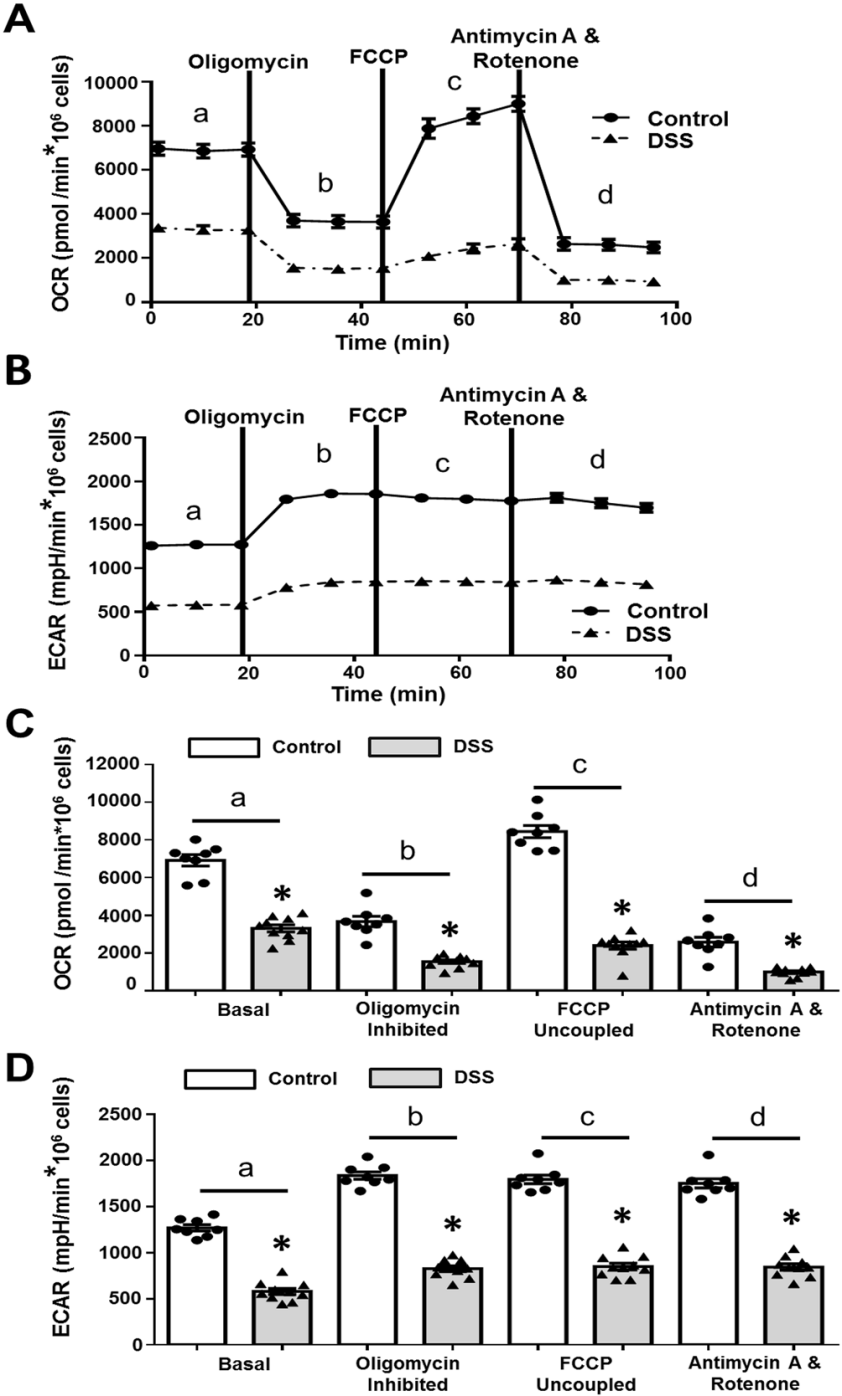
Results of Cell Mito Stress Protocol for IEC^4.1^ ± 2% DSS 24 h treatments using the Seahorse XF24 Analyzer. Graphs (**A**,**B**) represent tracings of oxygen consumption rates (OCR) and extracellular acidification rates (ECAR) respectively. Regions of metabolic significance are highlighted as (a) basal respiration, (b) Oligomycin-inhibited ATP-linked respiration, (c) FCCP-uncoupled maximal respiration, (d) complete mitochondrial inhibition. Graphs (**C**,**D**) summarize average OCR and ECAR values for well replicates during regions of metabolic interest. Data is presented as mean ± SEM (n = 8–10 replicates from 2 independent experiments); *Represents significant differences between the control group, p < 0.05; two-tailed independent t-tests for each metabolic region.

Results from the Seahorse XF mito stress test (Agilent Technologies Inc, USA) protocol for IEC^4.1^ under ± DSS treatment for 24 h are shown in Fig. 1. Representative tracings for OCR and extracellular acidification rates (ECAR) are shown in Fig. 1A and B respectively for both control and DSS treated cells. With the subtraction of non-mitochondrial respiration, OCR is relatively proportional to mitochondrial respiration, while ECAR is indicative of pH changes related to lactic acid production during glycolysis. Quantification of regions of metabolic significance are shown in Fig. 1C and D and include basal respiration, oligomycin-inhibited or ATP-linked respiration, carbonyl cyanide p-trifluoro-methoxyphenyl hydrazone (FCCP)-uncoupled or maximal respiration, and complete mitochondrial inhibition or non-mitochondrial respiration through the addition of antimycin A and rotenone. Examination of control and DSS treatments showed the latter to severely impair both OCR and ECAR, demonstrating the applicability of the technique.

### Optimization of metabolic assessment in the Oroboros O2k measurement system

Oroboros O2k (Oroboros Instruments, Austria) is a modular, high-resolution respirometer that analyzes oxygen consumption using polarographic oxygen sensors. The system allows for small biological samples (isolated mitochondria, cells, permeabilized tissues) to be analyzed within a closed chamber. Specific substrates and inhibitors allow basal, electron transport chain (ETC) complex-specific, and non-mitochondrial respiration to be determined in permeabilized cells (Supplementary Fig. 4, Table 2).

**Table 2:** Brief comparative evaluation of Seahorse XF24 Analyzer and Oroboros O2k by metrics relevant to potential investigators.

Property	Seahorse XF24 analyzer	Oroboros O2k
Sample capacity	High capacity multi-well to 96 well plate	Low capacity 2 chambers per machine
Sample	Intermittent stirring, cells added to microplates	Continuous stirring, cells suspended in solution
Machine cost	++++	++
Assay cost	+++	+
Co-culture	Yes	No
Protocol customization	Limited to 4 additions	Number of additions only limited by protocol duration
Sensitivity	Single calibration	Double calibration
Ease of use	+++	+
Additional protocols	Glycolysis	Reactive oxygen species, fluorometry

Another distinguishing feature of the system is that samples are constantly in motion (spinning chamber), ensuring uniform oxygen saturation and exposure to substrates and pharmacological drugs/inhibitors. For the Oroboros O2k, 1 × 10^6^ cell/mL cell suspension was selected based on manufacturer recommendation, as well as previously published literature and cell types^2,20^. To ensure mitochondrial access to cell-impermeable substrates (e.g. succinate, adenosine diphosphate), the cell membrane must be permeabilized using detergents, such as saponin or digitonin^16,21,22^. Cell permeabilization, allows substrates and inhibitors free access to mitochondria, thereby ensuring measurements are not affected by substrate availability. The concentration for permeabilization must be determined for each specific cell type because over titrating these detergents can lead to outer mitochondrial membrane (OMM) damage^16,23^. The optimal digitonin concentration for IEC^4.1^ was determined to be 3.24 µM (Supplementary Fig. 5).

For this platform, stable segments of normalized oxygen consumption were selected and used to calculate average rates of respiration for a given substrate, inhibitor, or uncoupler. Cell respiration was recorded in real-time at 37 °C. Specific to IEC^4.1^ cells, we defined stable respiration as within the range of ± 4 pmol/s. Proper machine calibration, cell characterization, and the optimization of both permeabilizing agent and substrate concentrations are critical for accurate measures. The concentrations of reagents used in each protocol are detailed in the methods section.

### Impact of DSS on Oroboros O2k oxygen consumption

**Figure 2:**
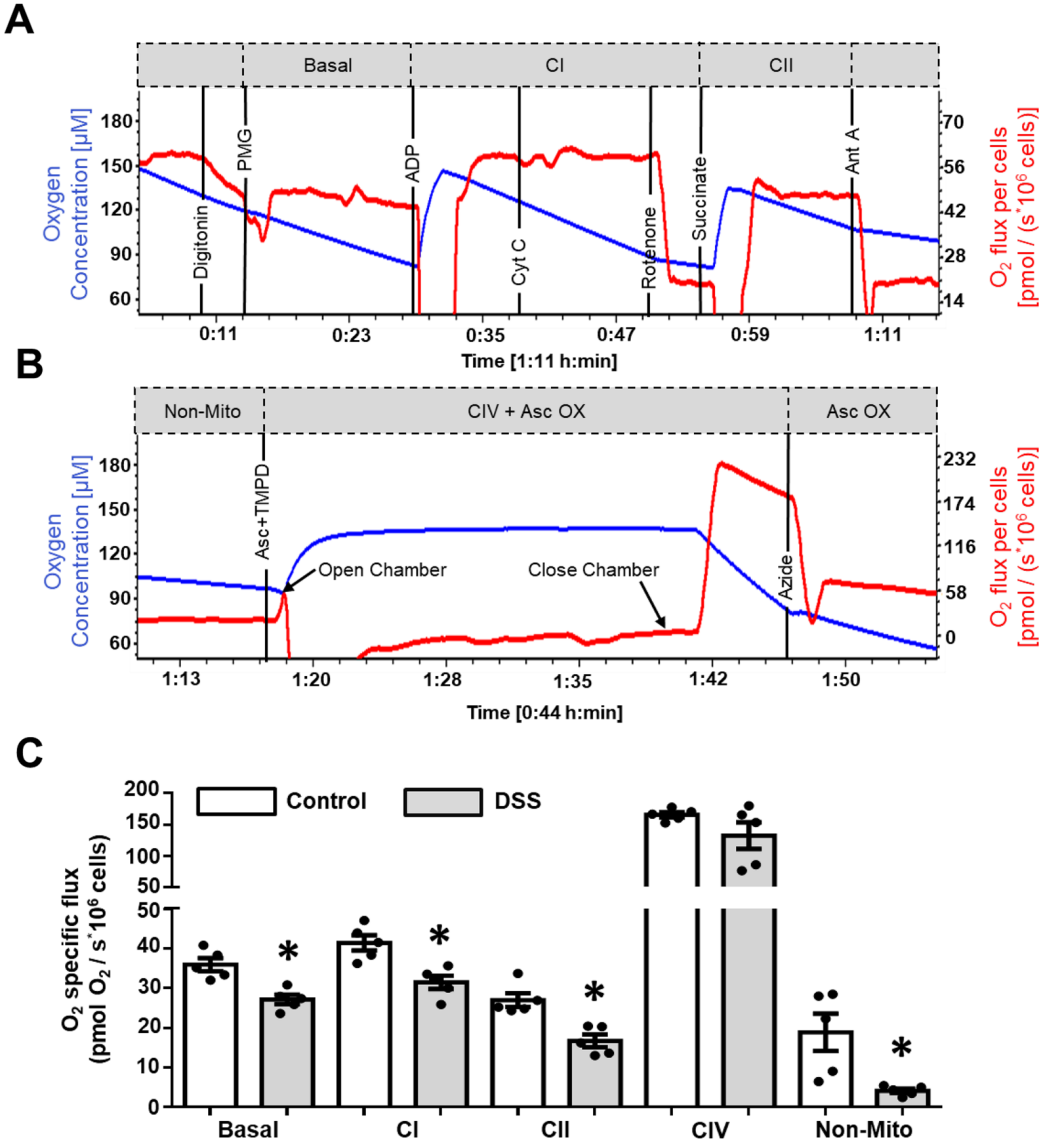
Results of Oroboros O2k for IEC^4.1^ ± 2% DSS 24 h treatments using the mitochondrial-specific complex protocol. (**A**,**B**) Oroboros O2k representative tracings of control IEC^4.1^ cells. (**C**) Mitochondrial-specific O_2_ flux during basal, mitochondrial complex linked (CI, CII, CIV) non-mitochondrial respiration (non-mito). Black vertical lines indicate additions of substrates/inhibitors/uncouplers., PMG: pyruvate (5 mM) + malate (2 mM) + glutamate (10 mM), ADP: adenosine diphosphate (2.5 mM), Cyt C: cytochrome c (10 µM), Rot: rotenone (0.5 µM), Succinate (10 mM), Ant A: antimycin A (2.5 µM), Asc + TMPD: ascorbate (2 mM) + TMPD (0.5 mM), Azide: sodium azide (≥ 100 mM). Gray hatched boxes represent different respiratory states: basal, complex I (CI), complex II (CII), non-mitochondrial respiration (Non-mito), and CIV + Asc Ox/Asc Ox (CIV). Data is presented as mean ± SEM (n = 5 replicates from 3 independent experiments; *Represents significant differences between the control group, p < 0.05; two-tailed independent t-tests for each respiratory state.

Oroboros O2k representative tracings and results obtained from permeabilized IEC^4.1^ ± DSS for 24 h are shown in Fig. 2. The timing of substrates and inhibitors and their impact on O_2_ flux, in addition to their related mitochondrial pathways, are shown in Fig. 2A and B. Note that 2B is a continuation of 2A and the figure was split into two panels to improve clarity. Briefly, basal respiration was determined by adding pyruvate, malate, and glutamate prior to the addition of ADP. Basal or leak respiration is the minimal amount of oxygen required to react with protons that passively leak from the intermembrane space as a consequence of proton leak. Within the context of permeabilized cells, leak respiration may be examined through use of reduced substrates (pyruvate, malate, glutamate) in medium containing inorganic phosphate (MiR05) and in the absence of adenylates like ADP^24^. The activity of ETC Complex I (CI) is examined by assessing the respiration of cells in the presence of CI-linked substrates (pyruvate, glutamate, malate) and subtracting the respiration obtained during CI inhibition by rotenone (Fig. 2A). This inhibition of CI by rotenone also allows Complex II (CII) activity to be identified without any influence of CI respiration. Before rotenone addition, cytochrome c is added to test for OMM damage, results are shown in Supplementary Fig. 6A. Damage to the OMM by permeabilizing agents can allow endogenous cytochrome c to leak from the mitochondria into the cytosol. Increases in O_2_ flux with exogenously supplied cytochrome c suggests OMM damage (Supplementary Fig. 7). Experiments that have a significant increase in O_2_ flux with cytochrome c should be carefully examined and possibly omitted when interpreting results.

Identification of CII activity follows a similar methodology to CI activity, with the subtraction of respiration in the presence of succinate as the substrate after the addition of an inhibitor like antimycin A. For CIV activity, *N,N,N′,N′*-tetramethyl-*p*-phenylenediamine (TMPD) is used to reduce cytochrome c, thereby acting as an artificial substrate. TMPD rapidly undergoes autoxidation with oxygen, so ascorbate is added before TMPD to maintain TMPD in a reduced state^16^. Due to the autoxidation of ascorbate and respiration of CIV, respiration increases dramatically and therefore requires the opening of the Oroboros O2k chambers to prevent cell hypoxia (Fig. 2B). Upon closure of the chambers, respiration will increase, eventually peak, and begin to linearly decline. To examine CIV, the respiration measurement after this peak must be subtracted by the respiration measurement after Complex IV (CIV) inhibition by sodium azide under similar time constraints (Fig. 2B). Aggregate results for control and DSS treatments are shown in Fig. 2C. DSS impaired CI, CII, and non-mitochondrial respiration. CIV was unaffected.

**Figure 3:**
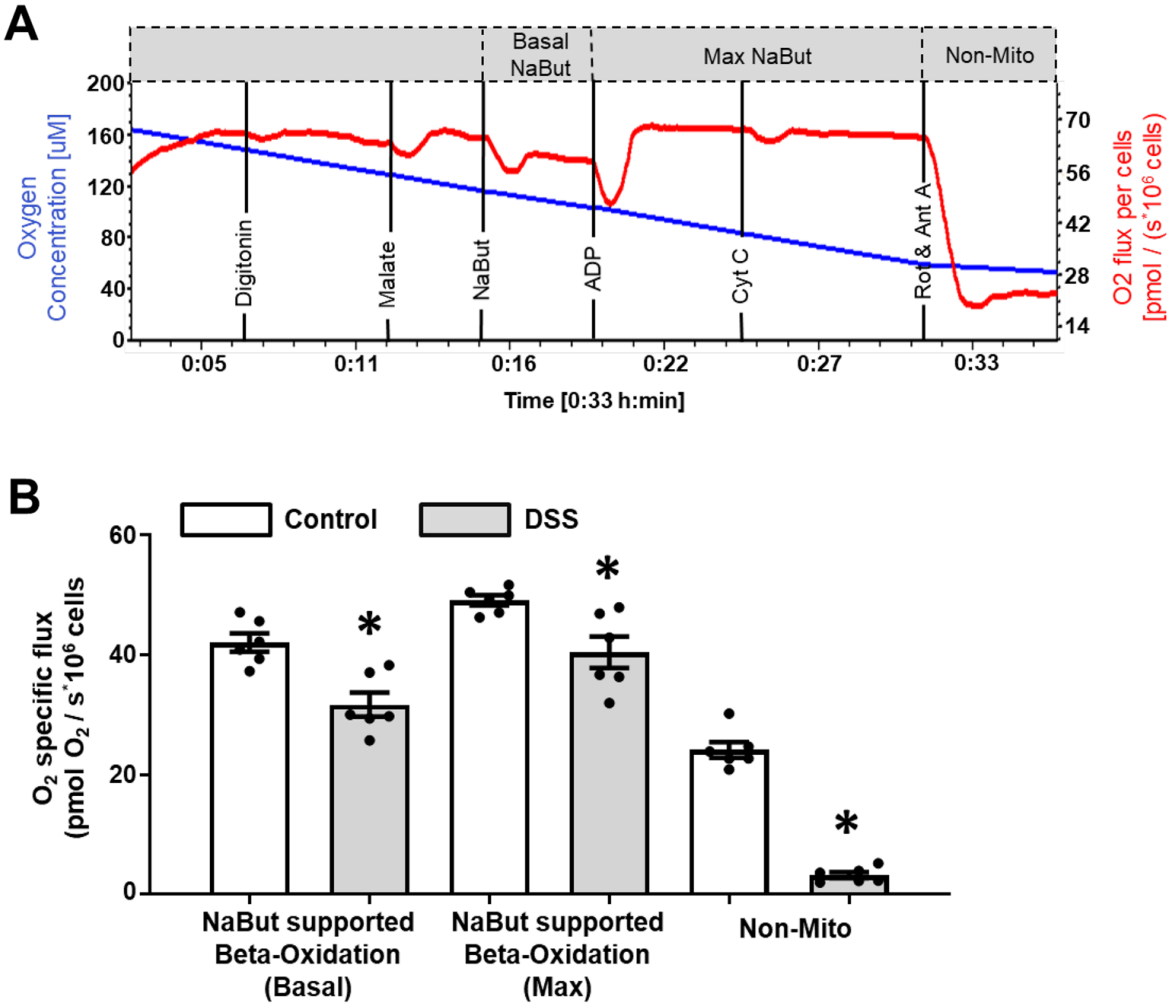
Examination of butyrate supported beta-oxidation in permeabilized IEC^4.1^ cells (10^6^ cells/mL) in the Oroboros O2k analyzer treated under control (white bars) or DSS (gray bars) conditions. (**A**) Oroboros O2k representative tracing of control IEC^4.1^ cells during the developed butyrate beta-oxidation protocol. Black vertical lines indicate additions of substrates/inhibitors/uncouplers. Malate (0.1 mM), NaBut: sodium butyrate (2 mM), ADP: adenosine diphosphate (2.5 mM), Cyt C: cytochrome c (10 µM), Rot: rotenone (0.5 µM), Ant A: antimycin A (2.5 µM). Gray hatched boxes represent different respiratory states: Basal NaBut (basal butyrate supported beta oxidation), Max NaBut (maximal butyrate supported beta oxidation), and non-mitochondrial respiration (Non-mito). (**B**) Mitochondrial-specific O_2_ flux responses after the addition of beta-oxidation linked substrates (malate, sodium butyrate) and mitochondrial inhibitors (non-mitochondrial respiration; rotenone, antimycin. Data is presented as mean ± SEM (n = 6 replicates from 2 independent experiments; *Represents significant differences between the control group, p < 0.05; two-tailed independent t-tests for each respiratory state.

Building on these results, we wanted to examine whether DSS could affect beta-oxidation of the short-chain fatty acid butyrate, a key colonocyte bioenergetic substrate^6^. Results and sample tracings are shown in Fig. 3. Briefly, beta-oxidation was assessed by adding fatty acid substrates and malate prior to the addition of other NADH-linked substrates. The addition of malate during beta-oxidation is necessary to prevent feedforward inhibition of beta-oxidation by the accumulation of short-chain acyl-CoAs and reduction in the acetyl-CoA pool^16^. The final concentration for butyrate (2 mM) was selected to ensure it served as a substrate, rather than as a potential signaling molecule^25,26^. Following this, additions of ADP ensure the saturation of adenylates for oxidative phosphorylation, thereby allowing maximal butyrate supported beta-oxidation to be measured (Fig. 3A). In the presence of malate and butyrate, basal respiration remained significantly lower for DSS-treated cells. This was also observed during maximal butyrate supported beta-oxidation after additions of ADP and during non-mitochondrial respiration after rotenone and antimycin A (Fig. 3B). As with the previous experiment, the lack of change between measurements before and after cytochrome c suggests that the OMM was not compromised after digitonin permeabilization and would not affect these results (Supplementary Fig. 6B)**.**

### Comparative evaluation of seahorse XF24 analyzer and Oroboros O2k

Although the results from permeabilized (Oroboros O2k) and non-permeabilized cells (Seahorse XF) cannot be directly compared, both platforms successfully reported the DSS insult to oxidative phosphorylation. To facilitate decision-making by investigators on the appropriate platform to employ, both the Seahorse XF24 Analyzer and Oroboros O2k are compared in Table 2.

## Discussion

The use of both the Oroboros O2k and Seahorse XF has been pivotal in helping to understand how mitochondrial function and the dysregulation of energy metabolism contributes to many disease states including IBD^14,15^. Moreover, mitochondrial function is now widely considered to be a potential target for clinical intervention^27^. In the present study, we developed and tested IEC^4.1^ protocols on two platforms, the Seahorse XF and the Oroboros O2k. As a proof of principle experiment, we utilized the Seahorse XF platform to examine the impact of 2% DSS exposure on mitochondrial respiration. Since DSS is an agent widely employed to induce murine colitis and has been previously reported to affect mitochondrial function, it presented a good agent for testing our protocols with the IEC^4.1^ cell line^28,29^.

Since the introduction of the Seahorse XF platform in 2006, there have been over 5000 publications exploring metabolic function in numerous tissues and samples. It has been used to examine isolated mitochondria^30^, immune cells^31^, cardiomyocytes^32^, and a non-transformed porcine jejunum-derived enterocyte cell line^33^ as well as many other sample types. The Seahorse XF sensors utilize porphyrin-based phosphors sensitive to both protons and oxygen. During measurement, emitted light excites the phosphors at multiple time points, and changes in the fluorescence emissions are related to OCR and ECAR^34^. In the Seahorse XF, administration of 2% DSS resulted in a global decline to both OCR and ECAR. For OCR, basal oxygen consumption was decreased by approximately half with DSS, while oligomycin-inhibited ATP-linked respiration, FCCP-uncoupled maximal respiration (also indicative of spare reserve capacity), and non-mitochondrial respiration were also reduced. Non-mitochondrial respiration is thought to be driven by processes outside the mitochondrial and cellular oxidative reactions not linked to energy metabolism^35^ was in the range of 10% of basal respiration, a number consistent with the literature and the cell type assessed^36,37^. Examination of ECAR, a measure of extracellular acidification was also measured during the assay. The increase in acidification following oligomycin is indicative of a shift towards glycolysis and anaerobic metabolism that is to be expected with the inhibition of mitochondrial oxidative phosphorylation.

Developed in 1994, the Oroboros O2k platform is available in 49 countries, and has been utilized in over 4000 publications to date. In comparison to the Seahorse XF, it is extremely cost-effective (no proprietary plates required), is lower throughput (2 chambers or samples at a time), but allows for greater control over experimental design, substrates, and inhibitors. Its main distinguishing features are that cells can be added as a suspension and stay constantly in motion, allowing uniform exposure to substrates and inhibitors. In contrast, the Seahorse XF cells are plated and although the plate is mixed, there is a range of diffusion phenomena that have the potential to affect results. An excellent review of such considerations and correction factors has been developed by Gerencser and colleagues^38^. Like the Seahorse XF, the Oroboros O2k has been used from many cell types but is also capable of examining small whole tissue sections like skeletal and cardiac muscle^22,39^.

The Oroboros O2k sensors are based on Leland Clark’s electrode technology first developed in 1954. These electrodes contained a membrane-covered oxygen electrode that had both the anode and cathode enclosed behind a non-conductive polyethylene membrane. In this set-up, the membrane (or sealed chamber) limited the permeability of oxygen making it possible to obtain quantitative measurements of oxygen tension in blood, solutions, and gases^40,41^. Our results from the Oroboros O2k demonstrated that 24 h exposure of cells to 2% DSS led to a decline in basal respiration, CI and CII activity, and non-mitochondrial respiration; while CIV activity was unaffected by DSS treatment. These findings are in agreement with previous studies showing CII to be lower in both the DSS mouse model of colitis as well as human biopsies obtained from macroscopically diseased colonic mucosa^11^. Like the Seahorse XF platform, maximal mitochondrial respiration and non-mitochondrial related respiration were again reduced with DSS treatment. Of these measures, maximal respiration, indicative of spare respiratory capacity is critical for cell survival under stressful conditions. Under normal, healthy conditions, IECs operate at basal levels that are sufficient to support general cellular functions including the maintenance of protein turnover and ion homeostasis. In this state, energy demands are continuously fine-tuned wherein energy demand is matched to energy generation, negating energy waste. However, with IBD and other insults such as DSS, energy demands escalate in an attempt to restore homeostasis. When demands cannot be met, mitochondria become stressed and undergo fission, documented by increases in reactive oxygen species production, inflammation, and a compromised intestinal barrier^27,42^. These insults further perpetuate tissue damage and disease progression. Given this, the greater the maximal, or spare respiratory capacity, the longer the tissue can theoretically maintain elevated levels of respiration, function, and even survival.

In addition to the abovementioned results, we also developed an assay to assess butyrate supported oxidation using the Oroboros 2 k, the primary energy substrate for colonic IECs. Butyrate is a short-chain fatty acid produced by the gut microbiota^43^. It serves as the preferred energy source for colonic IECs and can contribute > 80% of energetic demands^1^. For this reason, we substituted the octanoylcarnitine titrations in the Oroboros O2k SUIT-005 protocol for butyrate, making the protocol more physiologically relevant to colonic IECs. However, without the use of specific short chain fatty acid inhibitors, butyrate exclusive oxidation cannot be separated from contributions of endogenous substrates to oxygen flux. Unlike medium to long chain fatty acids, which can be inhibited by 2-mercaptoacetate and etomoxir respectively, there currently remains no widely adopted inhibitor for short chain fatty acids. This is an inherent limitation of this and other fatty acid assessments. Results demonstrated DSS significantly impaired basal and maximal butyrate supported oxidation. In line with this finding, Ahmad et al.^44^ showed that 4% DSS administration impaired murine isolated colonocyte butyrate oxidation by more than 50% compared to control animals.

In summary, although the results from permeabilized (Oroboros O2k) and non-permeabilized cells (Seahorse XF) cannot be directly compared, both platforms successfully reported the DSS insult to oxidative phosphorylation. This is important as reduced mitochondrial function has been shown to lead to loss of intestinal epithelial barrier function, a well-recognized feature of IBD^42,45^. However, elaborating and building on these discoveries has remained difficult due to a lack of established protocols and techniques for mitochondrial bioenergetic analysis. Attempting to bridge this gap and address issues with reproducibility, this study outlines detailed protocols, providing a user guide for conducting mitochondrial functional analysis using both the Seahorse XF and Oroboros O2k respirometric platforms.

## Materials and methods

### Cell culture

The murine ileal intestinal cell line IEC^4.1^^19^, was obtained from Dr. Li (Western University, London, ON). Cells were cultured in high glucose Dulbecco’s Modified Eagle Medium (4500 mg/L, 500 mL) supplemented with 50 mL of heat-denatured fetal bovine serum, 3 mL of 200 mM l-glutamine (1.07 mM), 5 mL of 1 M HEPES solution (8.91 mM), and 3 mL of 10 000U/mL penicillin–streptomycin solution (Gibco, U.S). Sub-culturing of cells occurred every 2–3 days when cells were 80–90% confluent as judged by phase-contrast microscopy with trypsin containing 0.5% EDTA (Gibco, U.S). Trypsinized cells were then diluted with culture media at a 1:2 ratio of trypsin to media. The cell solution was gently pipetted, collected, and centrifuged (Eppendorf 5810R Centrifuge, Germany), for 10 min at 361×*g* (1350 RPM, 17.7 cm arm radius). After centrifugation, the trypsin/media supernatant was siphoned off and the cell pellet was re-suspended in 3 mL of culture media. Cells were counted and seeded at 1 × 10^6^ cells in 100 × 20 mm culture dishes (Falcon, U.S). It is important to note that changing cell culture conditions (e.g. glucose deprivation, cell type, seeding density, etc.) will may require adjustments to measurement conditions including substrate concentrations.

### Seahorse XF measurement system

Seahorse XF (Seahorse XFe24 Analyzer, Agilent Technologies Inc, Santa Clara, CA) assays were conducted using the Seahorse XF Cell Mito Stress test kit, Seahorse XF calibrant, and cell culture microplates. Oxygen consumption rates (OCR) and extracellular acidification rates (ECAR) were measured using the Cell Mito Stress test procedure in prepared Seahorse XF assay media. Our protocol supplements the manufacturer's Cell Mito Stress protocol and uses 24 well cartridges with the Seahorse XF24 platform. The Seahorse XF assay media is required for the Cell Mito Stress protocol and must be prepared fresh before measurement. The assay media consists of 25 mM glucose, 4 mM L-glutamine, and 2 mM pyruvate, pH 7 with NaOH, adjusted at 30 °C.

#### Preparation of seahorse XF24 plates for cell mito stress analysis

1. Cells were seeded at 2 × 10^4^ cells per well (24 well cartridge format) and allowed to incubate (5% CO_2_, 37 °C) for 24 h before receiving treatments (control, 2% DSS) for an additional 24 h. Timing and number of cells will vary based on experimental design, treatments, and exposure times.
2. Perform machine calibration as per the manufacturer’s instructions.
3. Approximately 1 h before analysis, aspirate off culture media from the Seahorse plate with cells and add 1 mL of prepared Seahorse XF assay media. Carefully remove the Seahorse assay media, ensuring to not disturb the cell monolayer.
4. Add 500 μL of prepared Seahorse assay media to each well. Incubate the cell containing plate for 45 min (0% CO_2_, 37 °C).
5. Prepare and load chemicals into the ports of the calibrated cartridge:
  Port A add oligomycin (final concentration of 1.3μM).
  Port B add FCCP (final concentration of 0.5μM).
  Port C add antimycin A (final concentration of 1μM).
  Port D add rotenone (final concentration of 1μM).
6. Insert the sensor cartridge with the calibrant containing the bottom plate into the Seahorse XF24 machine for calibration. Once the machine is calibrated, remove the calibrant containing plate and add the cell containing plate to the machine. Begin measurement of the plate.
7. After the protocol has been run, gently add 100 µL of phosphate-buffered solution (PBS) (pH 7) to each well, collect and pipette off into a 2 mL microcentrifuge tube.
8. Add 50 µL of trypsin to each well and transfer cell culture plates to an incubator (5% CO_2_, 37 °C) for 5–8 min.
9. Add 100 µL of IEC^4.1^ culture media to each well, then pipette carefully to break clumps before adding to the previously filled PBS 2 mL microcentrifuge tube
10. Spin down cells at 1100RPM (244×*g*) for 10 min in a centrifuge (Eppendorf 5810R Centrifuge, Germany).
11. Pipette and discard the supernatant without disturbing the cell pellet, then re-suspend in 100 µL of IEC^4.1^ culture media.
12. Count both dead and live cells for the normalization of respirometric data.
13. Follow manufacturer's instructions regarding data interpretation.

### Oroboros O2K measurement system

#### MiR05 preparation and calibration

As per the manufacturer’s instructions, the mitochondrial respiratory solution-5 (MiR05) is required for calibration and analysis with the Oroboros O2k^23^. MiR05 is prepared using 0.5 mM EGTA, 3 mM MgCl_2_·6H_2_O, 20 mM, taurine, 10 mM KH_2_PO_4_, 20 mM HEPES, 1 g/L bovine serum albumin, 60 mM potassium-lactobionate, 110 mM sucrose, pH 7.1, adjusted to 30 °C. Once made, MiR05 should be sterilized using 0.45 µm filters and aliquoted into 50 mL tubes and stored at − 20 °C until needed. Following machine calibration^23^ experimentation can be initiated. Additional resources for troubleshooting and assistance with calculations can be found at https://www.bioblast.at/.

### Detailed protocols

#### Protocol 1: IEC^4.1^ cell preparation for Oroboros O2k respirometry

1. Prepare 6 × 10^6^ epithelial cells in 6 mL pre-warmed MiR05 medium using standard sterile cell culture procedures. Pipette gently to minimize cell clumps.
2. Set the Oroboros O2k chamber block temperature to 37.0 °C and stirrer speed to 750 rotation per minute (RPM). Next, calibrate the Oroboros O2k chambers by using the instructions outlined by the manufacturer^15^.
3. Gently add 2.3 mL of the 1 × 10^6^ cell/mL cell suspension to the calibrated Oroboros O2k chambers^23^. This cell number has been optimized for IEC^4.1^. The use of other cell types requires optimization before measurement^22^.
4. Slowly shift the stoppers downward into the “closed” position. Open DatLab (Oroboros Instruments, Austria) and observe the cells for 10 min or until respiration stabilizes (defined as O_2_ flux ≤ 4 pmol/s for IEC). Avoid leaving the IEC in the MiR05 for prolonged periods (≥ 1 h) prior to experimentation as they may become unresponsive to reagents.

#### Protocol 2: determining optimal digitonin concentration for IEC^4.1^ permeabilization

Since the Oroboros O2k contains spinning chambers, titration adjustments can easily be made and assessed for their impact on cell integrity or mitochondrial damage in real-time. This represents a key difference between the Oroboros O2k and the Seahorse XF platform. Here we outline the following steps to experimentally determine the correct concentration of digitonin for cell permeabilization. A complete list of chemicals and their respective actions on mitochondrial respiration are shown in Table 1 and Supplementary Fig. 4.

1. Add 2.3 mL of the 1 × 10^6^ cell/mL MiR05 cell suspension to the pre-calibrated Oroboros O2k chambers. Wait for 10–15 min or until respiration stabilizes at O_2_ flux ≤ 4 pmol/s.
2. Add 1 μL of 1 M rotenone (0.5 μM, Sigma Aldrich) into the Oroboros O2k chambers, wait 5 min.
3. Add 20 μL of 1 M succinate (10 mM, Sigma Aldrich) into the Oroboros O2k chambers and wait 5–10 min or until respiration stabilizes.
4. Add 10 μL of 0.5 M ADP (2.5 mM, Sigma Aldrich) into the Oroboros O2k chambers and wait 5–10 min or until respiration stabilizes.
5. Carefully titrate 0.2–0.4 μL of 10 mg/mL digitonin (Sigma Aldrich) and wait 5 min or until respiration stabilizes.
6. Continue stepwise addition of digitonin every 5 min until respiration no longer increases with subsequent additions. This is the concentration required for permeabilization. NOTE: Over titration of digitonin is also possible and leads to a continuous decline in cell respiration.
7. Record the concentration of digitonin required for permeabilization of cells and use that in future experiments. Both succinate and ADP additions are not expected to elicit increases in respiration since they are cell impermeable. However, the addition of digitonin will lead to increases in respiration due to increased cell membrane permeability and increased substrate access to mitochondria. Following this protocol, we determined that the optimum concentration for digitonin permeabilization of IEC^4.1^ cells was 3.24 µM.

#### Protocol 3: mitochondrial complex specific activity in permeabilized IEC^4.1^

To determine ETC complex activity, the Oroboros SUIT-008 protocol was used with minor modifications^46,47^. This protocol employs the addition of specific substrates and inhibitors listed in Table 1, that target defined points in mitochondrial energy generation pathways. IEC^4.1^ specific procedures are as follows.

1. Prepare a 1 × 10^6^ cell/mL cell suspension according to the steps outlined in Protocol 1. Wait for 10–15 min or until respiration stabilizes at O_2_ flux ≤ 4 pmol/s.
2. Add digitonin at the necessary concentration for permeabilizing cells based on experimentally determined results (0.8μL of 8.1 mM digitonin (3.24 μM) for IEC^4.1^ cells).
3. Add 5 μL of 2 M pyruvate (5 mM), 10 μL of 2 M glutamate (10 mM) and 10 μL of 400 mM malate (2 mM). These additions are done sequentially, one directly after another. Wait 10–15 min after these additions or until respiration stabilizes. O_2_ flux measurements taken after these additions will be used to calculate basal or leak respiration prior to the additions of any adenylates^24^.
4. Add 10 μL of 0.5 M ADP (2.5 mM) into the Oroboros O2k chambers and wait until respiration stabilizes. This allows the maximal capacities of mitochondrial complexes and substrates to be examined.
5. Add 5 μL of 4 mM cytochrome c (10 μM) into the Oroboros O2k chambers and wait until respiration stabilizes. This addition determines OMM integrity after digitonin permeabilization.
6. Add 1 μL of 1 M rotenone (0.5 μM) into the Oroboros O2k chambers and wait until respiration stabilizes. Rotenone inhibits ETC complex I (CI) and will allow CI activity to be determined by subtracting the difference of O_2_ flux before and after rotenone.
7. Add 20 μL of 1 M succinate (10 mM) into the Oroboros O2k chambers and wait until respiration stabilizes. Adding succinate after ETC CI inhibition with rotenone allows ETC CII respiration to be assessed, without interference by ETC CI respiration.
8. Add 1 μL of 5 mM antimycin A (2.5 μM) into the Oroboros O2k chambers and wait until respiration stabilizes. Antimycin A inhibits ETC CIII, resulting in the upstream inhibition of CII due to the inability to transfer electrons downstream. When combined with CI inhibition by rotenone, the ETC is completely inhibited. This allows non-mitochondrial respiration to be determined by measuring O_2_ flux after these additions.
9. Add 5 μL of 5 mM ascorbate (2 mM) followed immediately by 5 μL of 200 mM N*,N,N′,N′*-tetramethyl-*p*-phenylenediamine (TMPD, 0.5 mM). Open the chambers and record respiration for 20 min. Since TMPD is readily oxidized, ascorbate is added just before to serve as a sacrificial substrate to be oxidized instead, keeping TMPD in a reduced state. However, this means that the resulting O_2_ flux will include both ascorbate auto-oxidation and ETC CIV activity.
10. Close the chambers and record respiration for an additional 5 min.
11. Add 50 μL of 4 M sodium azide (≥ 100 mM) and record respiration for the next 10 min. Since sodium azide inhibits ETC CIV activity, the resulting O_2_ flux measured after sodium azide addition will correspond to O_2_ flux from ascorbate auto-oxidation. O_2_ flux for ascorbate auto-oxidation can then be subtracted from O_2_ flux measurements after chamber closure, but prior to the addition of sodium azide.

#### Protocol 4: measurement of butyrate supported beta-oxidation in IEC^4*.1*^

As butyrate is the primary substrate metabolized by colonocytes, a protocol to assess the oxidation of this short-chain fatty acid was developed based on a modification of the SUIT-005 protocol^48^. IEC^4.1^ specific procedures are as follows:

1. Prepare a 1 × 10^6^ cell/mL cell suspension according to the steps, load into the chamber, and wait for 10–15 min or until respiration stabilizes (Protocol 1).
2. Permeabilize cells by adding digitonin until the experimentally determined concentration is reached (0.8 μL of 8.1 mM digitonin (3.24 μM) for IEC^4.1^ cells).
3. Add 4 μL of 50 mM malate (0.1 mM) into the Oroboros O2k chambers and wait until respiration stabilizes. Typically at this concentration and when paired with a fatty acid, malate is utilized predominantly to regenerate acetyl-CoA for the continuation of beta-oxidation, rather than NADH production during the tricarboxylic acid (TCA, or Krebs) cycle^49^.
4. Add 4 μL of 1 M sodium butyrate (2 mM) into the Oroboros O2k chambers and wait until respiration stabilizes. Similar to Protocol 3, the addition of malate and butyrate without adenylates limits oxidative phosphorylation to just leak or basal respiration of butyrate supported beta-oxidation^24^.
5. Add 10 μL of 0.5 M ADP (2.5 mM) into the Oroboros O2k chambers and wait 5–10 min or until respiration stabilizes. Similar to Protocol 3, ADP is added to ensure oxidative phosphorylation is not limited by the lack of adenylates, thereby allowing maximal butyrate supported beta-oxidation to be measured.
6. Add 5 μL of 4 mM cytochrome c (10 μM) into the Oroboros O2k chambers and wait until respiration stabilizes.
7. Add 1 μL of 1 M rotenone (0.5 μM) and 1 μL of 5 mM antimycin A (2.5 μM) into the Oroboros O2k chambers and wait until respiration stabilizes. By ending the protocol with these agents, non-mitochondrial respiration can be measured and subtracted from other measurements.

### Calculations and statistics

Experimental data is presented as means ± standard error of the mean, with individual dots representing chamber replicates from independent experiments. Two-tailed, independent t-tests were used for comparisons of samples during different respiratory states. Statistically significant results were determined if p < 0.05. Statistical analysis was performed with GraphPad 6 (GraphPad, La Jolla, CA). OCR, ECAR, and O_2_ flux were normalized to cell concentrations of 10^6^ cells/mL. For the Oroboros O2k data, some DSS data points were part of a larger study and have been previously published^27^ and are shown here to provide context to the outlined respirometric methods. For each platform, it is recommended that manufacturer software be employed for data analysis (DatLab for Oroboros O2k and Wave Desktop Software for Seahorse XF).

## Supplementary Information

Supplementary Figures.

## Data Availability

Data are available upon reasonable request up until 1 year after publication.

## References

[1] Roediger WE (1982). Utilization of nutrients by isolated epithelial cells of the rat colon. Gastroenterology.

[2] Janssen Duijghuijsen LM, Grefte S, de Boer VCJJ, Zeper L, van Dartel DAMM, van der Stelt I (2017). Mitochondrial ATP depletion disrupts Caco-2 monolayer integrity and internalizes claudin 7. Front. Physiol..

[3] Bär F, Bochmann W, Widok A, von Medem K, Pagel R, Hirose M (2013). Mitochondrial gene polymorphisms that protect mice from colitis. Gastroenterology.

[4] Roediger WE (1980). The colonic epithelium in ulcerative colitis: an energy-deficiency disease?. Lancet.

[5] Chapman MA, Grahn MF, Boyle MA, Hutton M, Rogers J, Williams NS (1994). Butyrate oxidation is impaired in the colonic mucosa of sufferers of quiescent ulcerative colitis. Gut.

[6] Duffy MM, Regan MC, Ravichandran P, O’Keane C, Harrington MG, Fitzpatrick JM (1998). Mucosal metabolism in ulcerative colitis and Crohn’s disease. Dis. Colon. Rectum.

[7] Yuksel M, Ates I, Kaplan M, Arikan MF, Ozin YO, Kilic ZMY (2017). Is oxidative stress associated with activation and pathogenesis of inflammatory bowel disease?. J. Med. Biochem..

[8] Boyapati RK, Dorward DA, Tamborska A, Kalla R, Ventham NT, Doherty MK (2018). Mitochondrial DNA is a pro-inflammatory damage-associated molecular pattern released during active IBD. Inflamm. Bowel Dis..

[9] Sharma D, Kanneganti T-D (2017). Inflammatory cell death in intestinal pathologies. Immunol. Rev..

[10] Shekhawat PS, Srinivas SR, Matern D, Bennett MJ, Boriack R, George V (2007). Spontaneous development of intestinal and colonic atrophy and inflammation in the carnitine-deficient jvs (OCTN2-/-) mice. Mol. Genet. Metab..

[11] Santhanam S, Rajamanickam S, Motamarry A, Ramakrishna BS, Amirtharaj JG, Ramachandran A (2012). Mitochondrial electron transport chain complex dysfunction in the colonic mucosa in ulcerative colitis. Inflamm. Bowel Dis..

[12] Roediger WEW, Nance S (1986). Metabolic induction of experimental ulcerative colitis by inhibition of fatty acid oxidation. Br. J. Exp. Pathol..

[13] Ni J, Chen SF, Hollander D (1996). Effects of dextran sulphate sodium on intestinal epithelial cells and intestinal lymphocytes. Gut.

[14] Mancini NL, Rajeev S, Jayme TS, Wang A, Keita ÅV, Workentine ML (2021). Crohn’s disease pathobiont adherent-invasive *E coli* disrupts epithelial mitochondrial networks with implications for gut permeability. CMGH.

[15] McKay DM, Mancini NL, Shearer J, Shutt T (2020). Perturbed mitochondrial dynamics, an emerging aspect of epithelial-microbe interactions. Am. J. Physiol. Gastrointest. Liver Physiol..

[16] Doerrier C, Sumbalova Z, Krumschnabel G, Gnaiger E (2016). SUIT reference protocol for OXPHOS analysis by high-resolution respirometry. Mitochondr. Physiol. Netw..

[17] Bekebrede AF, Keijer J, Gerrits WJJ, de Boer VCJ (2021). Mitochondrial and glycolytic extracellular flux analysis optimization for isolated pig intestinal epithelial cells. Sci. Rep..

[18] Grauso M, Lan A, Andriamihaja M, Bouillaud F, Blachier F (2019). Hyperosmolar environment and intestinal epithelial cells: Impact on mitochondrial oxygen consumption, proliferation, and barrier function in vitro. Sci. Rep..

[19] Li XC, Jevnikar AM, Grant DR (1997). Expression of functional ICAM-1 and VCAM-1 adhesion molecules by an immortalized epithelial cell clone derived from the small intestine. Cell. Immunol..

[20] Djafarzadeh S, Jakob SM (2017). High-resolution respirometry to assess mitochondrial function in permeabilized and intact cells. J. Vis. Exp..

[21] Kuznetsov AV, Veksler V, Gellerich FN, Saks V, Margreiter R, Kunz WS (2008). Analysis of mitochondrial function in situ in permeabilized muscle fibers, tissues and cells. Nat. Protoc..

[22] Hughey CC, Hittel DS, Johnsen VL, Shearer J (2010). Respirometric oxidative phosphorylation assessment in saponin-permeabilized cardiac fibers. J. Vis. Exp..

[23] Gnaiger E (2020). O2k quality control 1: Polarographic oxygen sensors and accuracy of calibration. Mitochondr. Physiol. Netw..

[24] Gnaiger E (2009). Capacity of oxidative phosphorylation in human skeletal muscle: New perspectives of mitochondrial physiology. Int. J. Biochem. Cell Biol..

[25] Andriamihaja M, Chaumontet C, Tome D, Blachier F (2009). Butyrate metabolism in human colon carcinoma cells: Implications concerning its growth-inhibitory effect. J. Cell Physiol..

[26] Davie JR (2003). Inhibition of histone deacetylase activity by butyrate. J. Nutr..

[27] Mancini NL, Goudie L, Xu W, Sabouny R, Rajeev S, Wang A (2020). Perturbed mitochondrial dynamics is a novel feature of colitis that can be targeted to lessen disease. CMGH.

[28] Diaz-Granados N, Howe K, Lu J, McKay DM (2000). Dextran sulfate sodium-induced colonic histopathology, but not altered epithelial ion transport, is reduced by inhibition of phosphodiesterase activity. Am. J. Pathol..

[29] Damiani CR, Benetton CAF, Stoffel C, Bardini KC, Cardoso VH, Di Giunta G (2007). Oxidative stress and metabolism in animal model of colitis induced by dextran sulfate sodium. J. Gastroenterol. Hepatol..

[30] Iuso A, Repp B, Biagosch C, Terrile C, Prokisch H (2017). Assessing mitochondrial bioenergetics in isolated mitochondria from various mouse tissues using Seahorse XF96 analyzer. Methods Mol. Biol..

[31] Kramer PA, Prichard L, Chacko B, Ravi S, Turner Overton E, Heath SL (2015). Inhibition of the lymphocyte metabolic switch by the oxidative burst of human neutrophils. Clin. Sci..

[32] Ali Pour P, Kenney MC, Kheradvar A (2020). Bioenergetics consequences of mitochondrial transplantation in cardiomyocytes. J. Am. Heart Assoc..

[33] Tan B, Xiao H, Li F, Zeng L, Yin Y (2015). The profiles of mitochondrial respiration and glycolysis using extracellular flux analysis in porcine enterocyte IPEC-J2. Anim. Nutr..

[34] Koopman M, Michels H, Dancy BM, Kamble R, Mouchiroud L, Auwerx J (2016). A screening-based platform for the assessment of cellular respiration in *Caenorhabditis elegans*. Nat. Protoc..

[35] Herst PM, Tan AS, Scarlett D-JG, Berridge MV (2004). Cell surface oxygen consumption by mitochondrial gene knockout cells. Biochim. Biophys. Acta Bioenerg..

[36] Shen J, Khan N, Lewis LD, Armand R, Grinberg O, Demidenko E (2003). Oxygen consumption rates and oxygen concentration in molt-4 cells and their mtDNA depleted (ρ0) mutants. Biophys. J..

[37] Chandel NS, Maltepe E, Goldwasser E, Mathieu CE, Simon MC, Schumacker PT (1998). Mitochondrial reactive oxygen species trigger hypoxia-induced transcription. Proc. Natl. Acad. Sci. USA.

[38] Gerencser AA, Neilson A, Choi SW, Edman U, Yadava N, Oh RJ (2009). Quantitative microplate-based respirometry with correction for oxygen diffusion. Anal. Chem..

[39] Hughey CC, James FD, Ma L, Bracy DP, Wang Z, Wasserman DH (2014). Diminishing impairments in glucose uptake, mitochondrial content, and ADP-stimulated oxygen flux by mesenchymal stem cell therapy in the infarcted heart. Am. J. Physiol. Cell Physiol..

[40] Severinghaus JW, Astrup PB (1986). History of blood gas analysis. IV. Leland Clark’s oxygen electrode. J. Clin. Monit..

[41] Wolf R, Granger D, Taylor Z (1953). Continuous recording of blood oxygen tensions by polarography. J. Appl. Physiol..

[42] Wang A, Keita ÅV, Phan V, McKay CM, Schoultz I, Lee J (2014). Targeting mitochondria-derived reactive oxygen species to reduce epithelial barrier dysfunction and colitis. Am. J. Pathol..

[43] Vital M, Howe AC, Tiedje JM (2014). Revealing the bacterial butyrate synthesis pathways by analyzing (meta)genomic data. MBio.

[44] Ahmad MS, Krishnan S, Ramakrishna BS, Mathan M, Pulimood AB, Murthy SN (2000). Butyrate and glucose metabolism by colonocytes in experimental colitis in mice. Gut.

[45] Nazli A, Yang PC, Jury J, Howe K, Watson JL, Söderholm JD (2004). Epithelia under metabolic stress perceive commensal bacteria as a threat. Am. J. Pathol..

[46] Huete-Ortega, M., Iglesias-Gonzalez, J. & Gnaiger, E. *Reference: A: for cells-permeabilized cells. SUIT Number D025_ce1;1Dig;1PM;2D;2c;3G;4S;5U;6Rot;7Ama;8AsTm;9Azd*. https://www.bioblast.at/index.php/SUIT-008_O2_ce-pce_D025. Accessed 25 Aug 2020.

[47] Lemieux H, Blier PU, Gnaiger E (2017). Remodeling pathway control of mitochondrial respiratory capacity by temperature in mouse heart: Electron flow through the Q-junction in permeabilized fibers. Sci. Rep..

[48] Gnaiger, E. & Iglesias-Gonzalez, J. *Oroboros SUIT-005 Protocol 2020*. https://bioblast.at/index.php/SUIT-005.

[49] Doerrier C, Garcia-Souza LF, Krumschnabel G, Wohlfarter Y, Mészáros AT, Gnaiger E (2018). High-resolution fluorespirometry and oxphos protocols for human cells, permeabilized fibers from small biopsies of muscle, and isolated mitochondria. Methods Mol. Biol..

