# Supplementary material for: Impact of experimental colitis on mitochondrial bioenergetics in intestinal epithelial cells
Source: Sci Rep. 2022 May 6;12:7453. doi: 10.1038/s41598-022-11123-w (PMC9076608; doi:10.1038/s41598-022-11123-w)
Supplement: Supplementary file 1 — Supplementary Figures. [file 41598_2022_11123_MOESM1_ESM.docx]

*Data Supplement*

**Impact of experimental colitis on mitochondrial bioenergetics in intestinal epithelial cells**

Luke Goudie^1^, Nicole L. Mancini^2^, Timothy E. Shutt^3^, Graham P. Holloway^4^, Chunlong Mu^3^, Arthur Wang^2^, Derek M. McKay^2^, & Jane Shearer^1,3*^

^1^Department of Biomedical Engineering, Schulich School of Engineering, University of Calgary, Alberta, Canada

^2^Gastrointestinal Research Group and Inflammation Research Network, Department of Physiology and Pharmacology, Calvin, Joan and Phoebe Snyder Institute for Chronic Diseases, Cumming School of Medicine, University of Calgary, Alberta, Canada

^3^Alberta Children’s Hospital Research Institute, Hotchkiss Brain Institute, Departments of Medical Genetics and Biochemistry and Molecular Biology, Cumming School of Medicine, University of Calgary, Alberta, Canada

^4^Department of Human Health and Nutritional Sciences, University of Guelph, Ontario, Canada

**Running Title:** Intestinal Epithelial Bioenergetics

***Corresponding Author:** Jane Shearer. Alberta Children’s Hospital Research Institute. 3330 Hospital Drive NW. HMRB 228. University of Calgary, Calgary, Alberta, Canada. Email: jshearer@ucalgary.ca


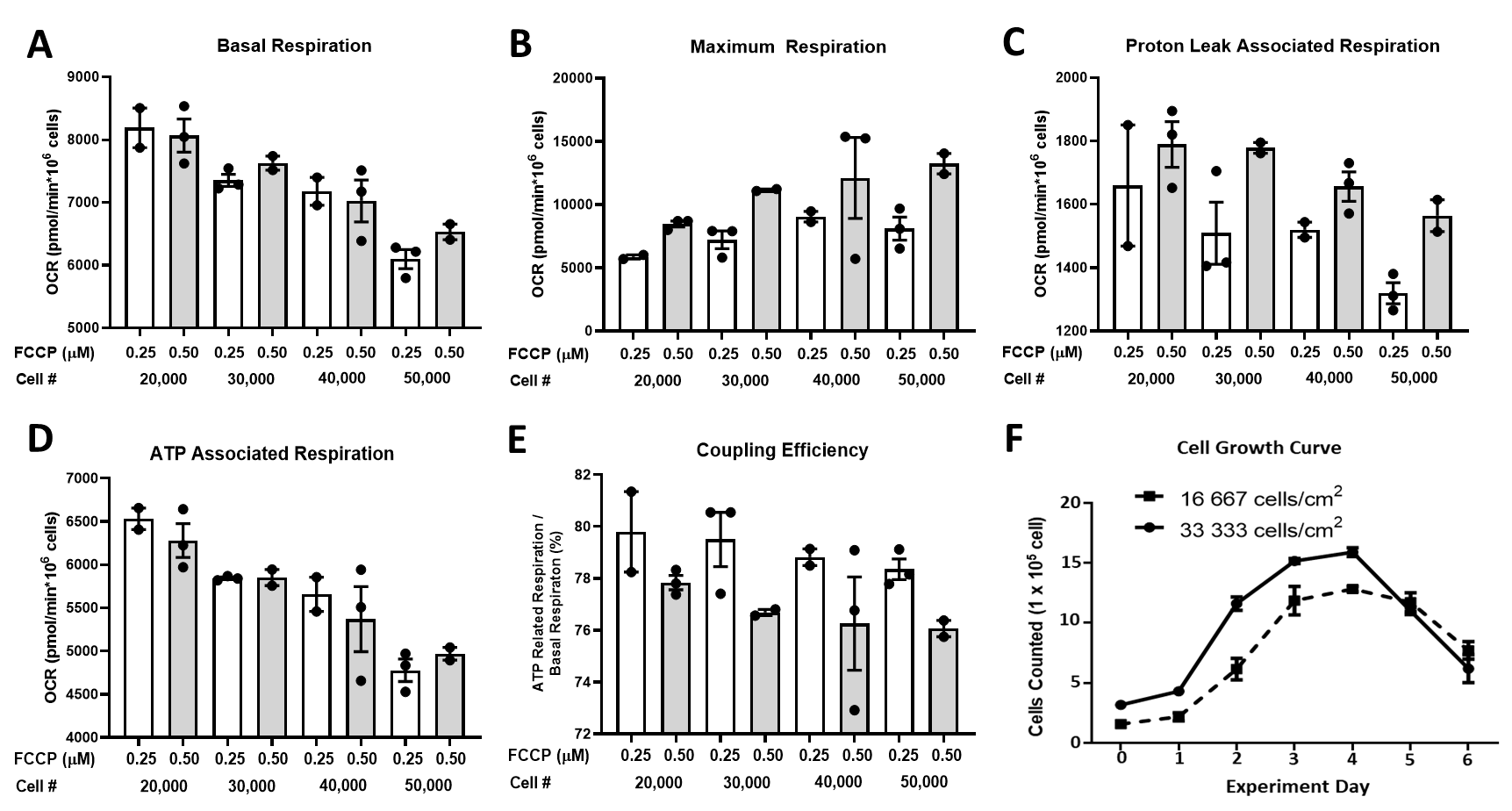


**Supplementary Figure 1:** IEC^4.1^ experimental growth and mitochondrial energetic results for optimizing cell density in Seahorse XF experiments (2 x 10^4^ – 5 x 10^4^ cells/well). Graphs A-E represent summary results of metabolically significant points of interest: A) Basal respiration, B) Maximal respiration C) Proton-Leak associated respiration, D) ATP-associated respiration and E) Coupling efficiency for varying cell densities (2 x 10^4^ – 5 x 10^4^ cells/well) and FCCP concentrations (0.25-0.5µM). Graph F shows results for IEC^4.1^ cell growth. Data is presented as mean ± SEM (n = 3 replicates from 1 independent experiment).


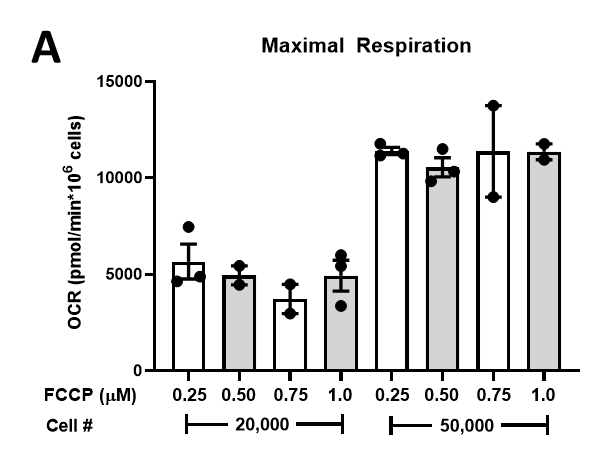


**Supplementary Figure 2:** Optimization of FCCP concentrations for Seahorse XF using IEC^4.1^ cells under varying cell densities (2 x 10^4^ & 5 x 10^4^ cells/well) and FCCP concentrations (0.25-0.5µM). Graph A shows maximal oxygen consumption rates (OCR) measurements of IEC^4.1^ cells (2 x 10^4^ & 5 x 10^4^ cells/well) after titrations of different FCCP concentrations (0.25-0.5 µM). Data is presented as mean ± SEM (n = 3 replicates from 1 independent experiment).


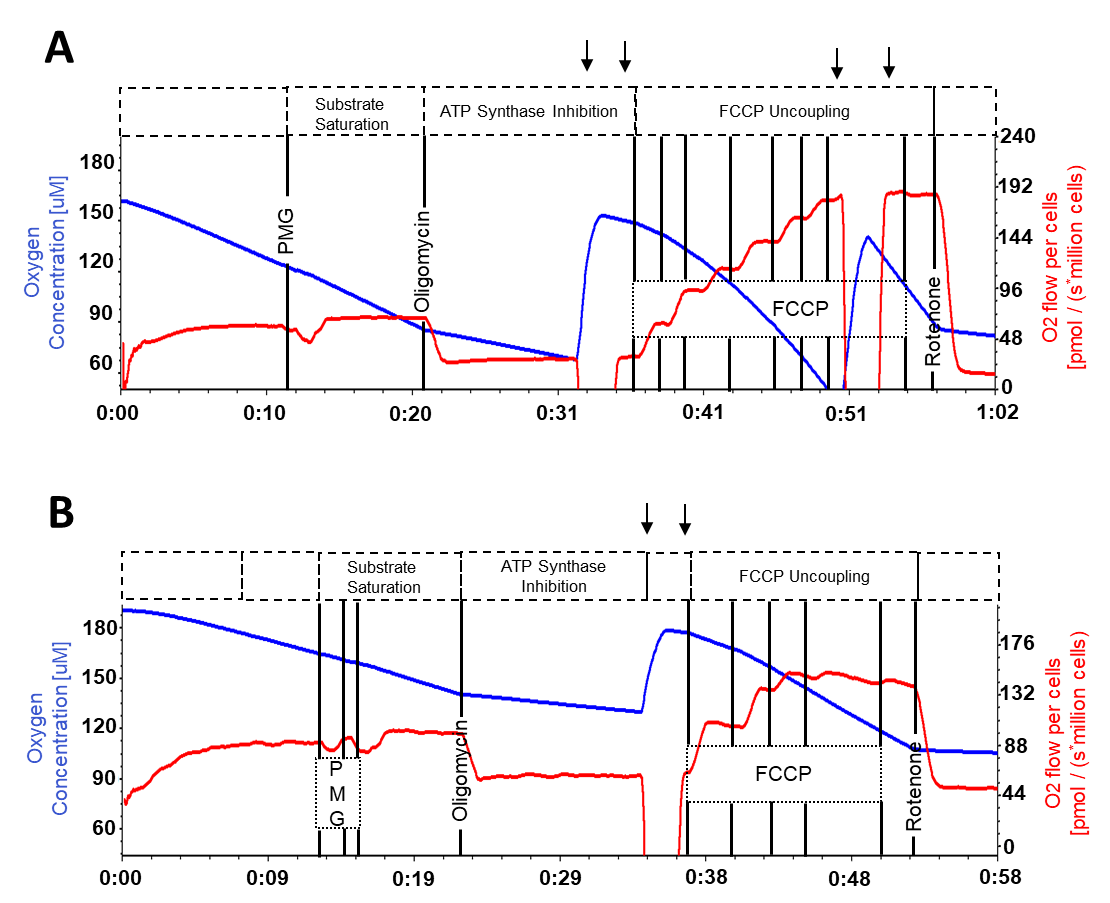


**Supplementary Figure 3.** Oroboros O2k representative tracing for optimization of FCCP concentrations using IEC^4.1^ cells under control and DSS conditions (10^6^ cells/mL). Black vertical lines indicate additions of substrates/inhibitors/uncouplers. Figure A is a representative tracing of control IEC^4.1^ cells, while Figure B is a representative tracing of DSS treated IEC^4.1^ cells. PMG (Pyruvate (5mM), Malate (2mM), Glutamate (10mM)), Oligomycin (2.5μM), and Rotenone (0.5μM). FCCP serial titrations ranged from 0.1-0.3 µL with Figure A FCCP concentrations being: 0.10, 0.20, 0.25, 0.30, 0.40, 0.50, 0.60, and 0.70μM and Figure B as: 0.15, 0.30, 0.40, 0.45 and 0.50μM. Gray hatched box labeled: substrate saturation with pyruvate+malate+glutamate, ATP synthase inhibition by oligomycin and respiratory uncoupling with progressively higher concentrations of FCCP. Downward arrows indicate opening (1^st^ arrow) and closing (2^nd^ arrow) of O2k chambers for re-oxygenation.

**
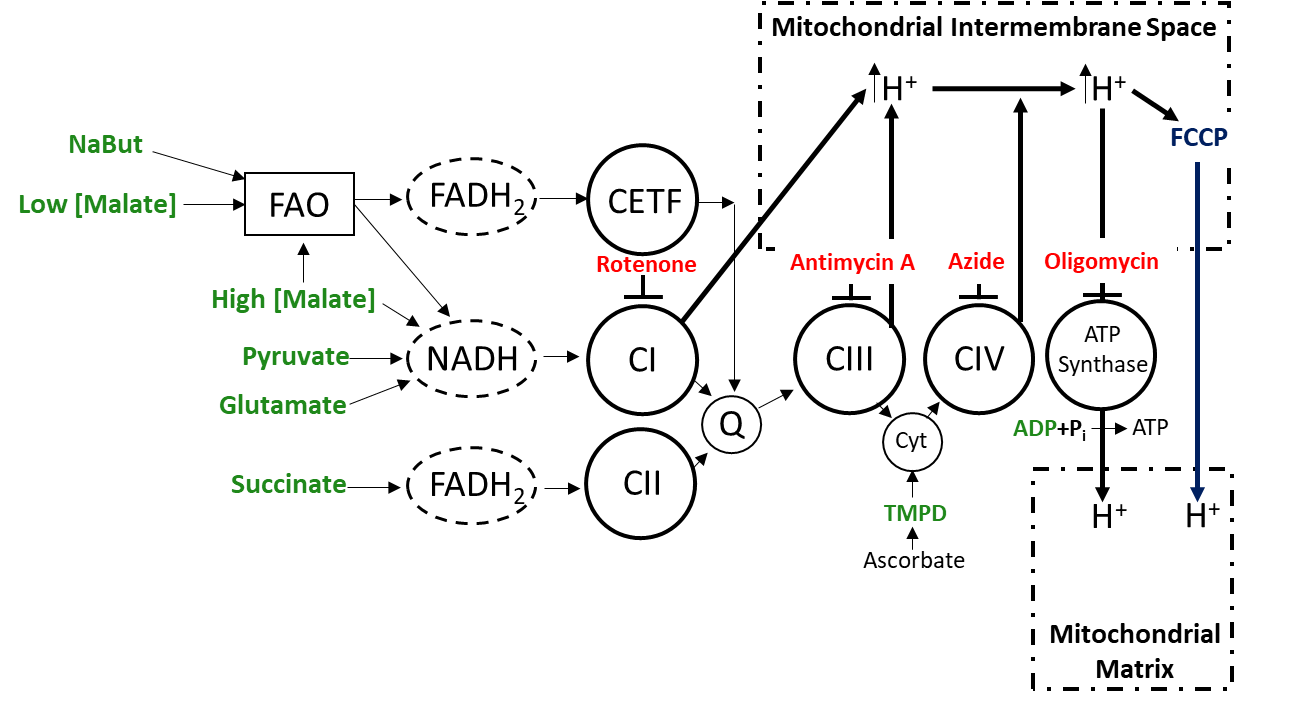
**

**Supplementary Figure 4.** Flow diagram of the interactions for substrate (green), inhibitor (red), and uncoupler (blue) additions with their relevant mitochondrial pathways and complexes. NaBut: butyrate, FAO: fatty acid oxidation, FADH_2_: flavin adenine dinucleotide, NADH: nicotinamide adenine dinucleotide, CETF: electron transferring flavoprotein complex, CI: mitochondrial complex I, CII: mitochondrial complex II Q: Coenzyme Q_10_, CIII: mitochondrial complex III, CIV: mitochondrial complex IV Cyt: cytochrome C, TMPD: N,N,N′,N′-tetramethyl-p-phenylenediamine, ADP: adenosine diphosphate, P_i_: inorganic phosphate, ATP: adenosine triphosphate, FCCP: carbonyl cyanide p-trifluoro-methoxyphenyl. Dashed boxes indicate compartments of mitochondria.

**
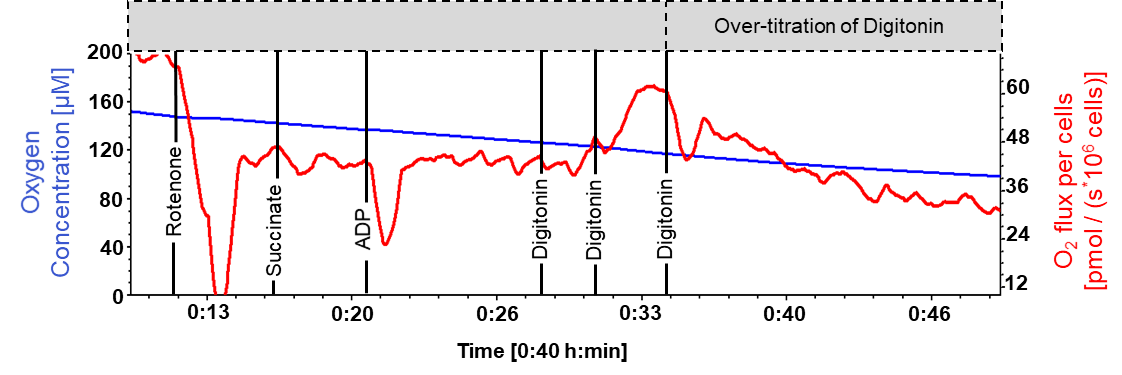
**

**Supplementary Figure 5.** Oroboros O2k representative tracing for digitonin optimization protocol in IEC^4.1^ cells (10^6^ cells/mL). Black vertical lines indicate additions of substrates/inhibitors/uncouplers. Rotenone (0.5µM), Succinate (10mM) ADP: adenosine diphosphate (2.5mM), Digitonin (1.62μM, 3.24μM, 4.05μM). Gray hatched box labeled: Over-titration of digitonin, highlights decline in respiration after too many titrations of digitonin.

**A**

**B**

**
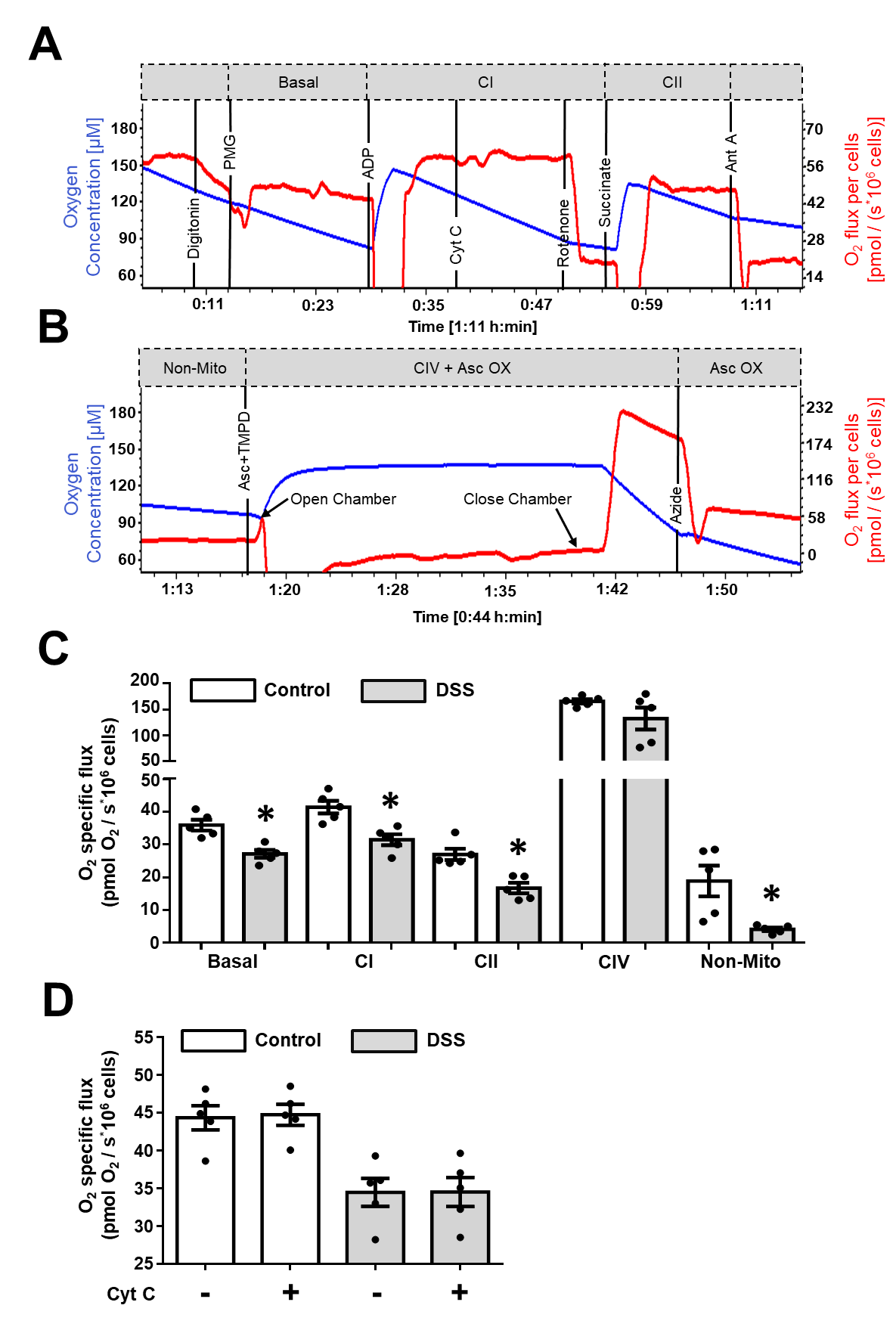

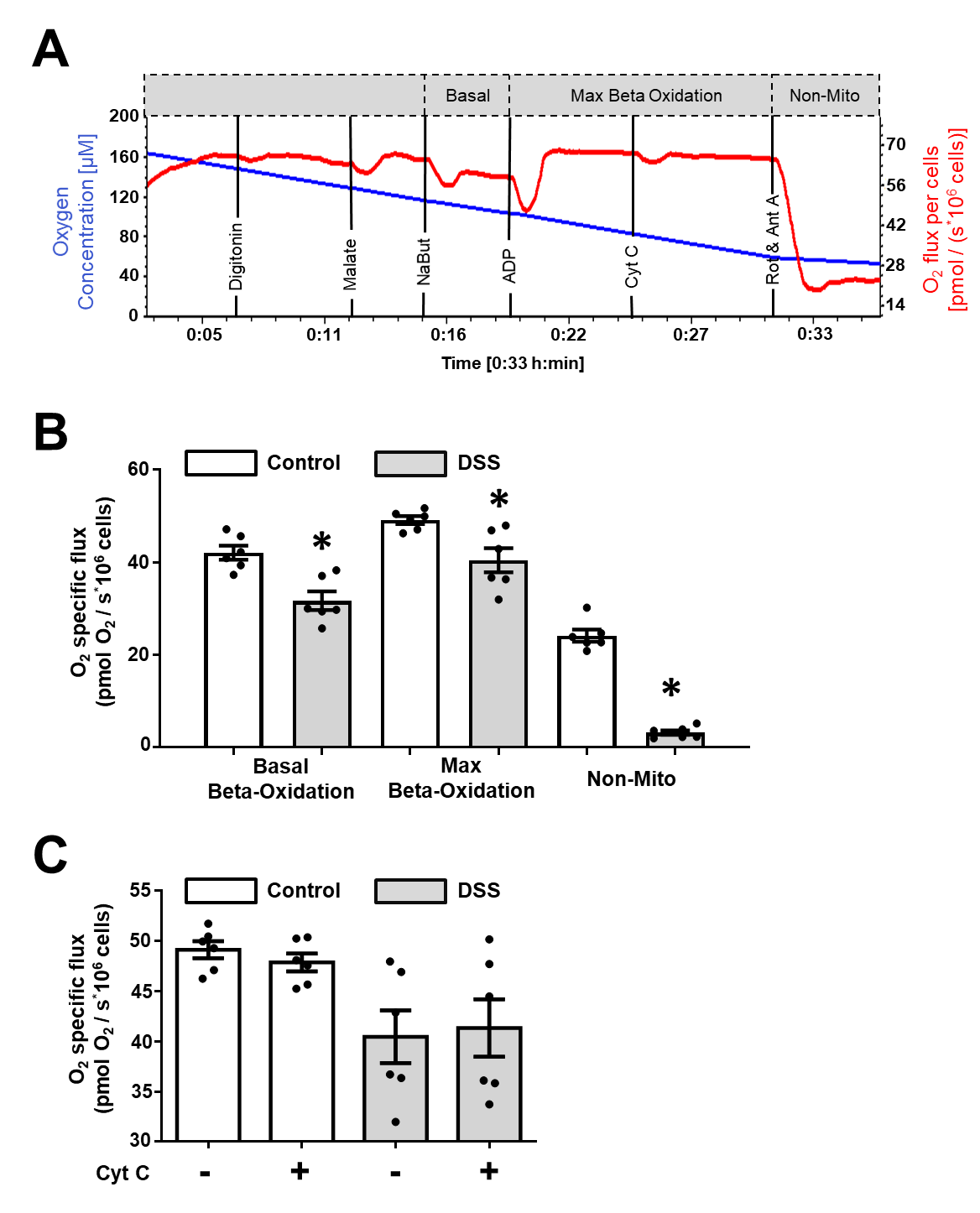
**

**Supplementary Figure 6.**  Mitochondrial-specific O_2_ flux of IEC^4.1^ cells during the outer mitochondrial membrane test using cytochrome c (Cyt C) for the (A) mitochondrial complex activity and (B) beta-oxidation protocols using the Oroboros O2k system.

**
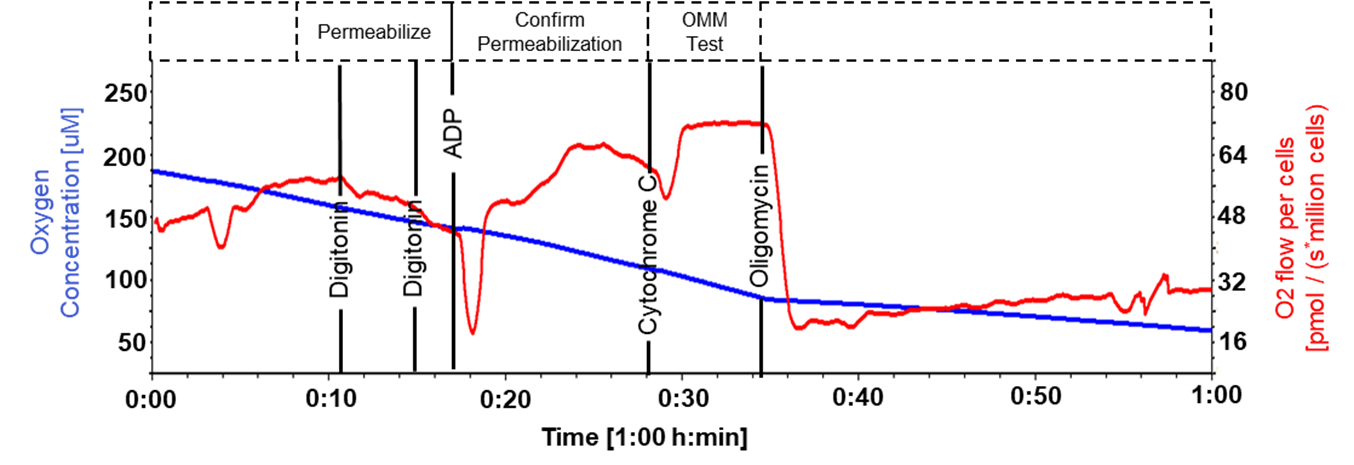
**

**Supplementary Figure 7.** Oroboros O2k representative tracing demonstrating over-titration of digitonin and confirmation of over-permeabilization by use of cytochrome C in IEC 4.1 cells. Black vertical lines indicate additions of substrates and inhibitors: Digitonin (8.10μM, 9.72μM), ADP: adenosine diphosphate (2.5mM), cytochrome C (10μM) and oligomycin (2.5μM). Gray hatched box labeled: Permeabilization of cells by digitonin, confirmation of permeabilization by ADP and test of outer mitochondrial integrity (OMM) by cytochrome c. Increased respiration after cytochrome c suggests OMM has been compromised from over-titrating digitonin.
